# Retrospective analysis of urethral anastomosis with ancillary maneuvers and intraoperative biaxial defect measurements to achieve a tension free guidance system for redo PFUDD treatment

**DOI:** 10.1186/s12894-024-01456-1

**Published:** 2024-04-09

**Authors:** Kaile Zhang, Meng Liu, Tiantian Wang, Qiang Fu

**Affiliations:** 1 The Department of Urology, affiliated Sixth People’s Hospital, Shanghai Jiaotong University School of medicine, Shanghai, 200233 China; 2 Shanghai Eastern Institute of Urologic Reconstruction, Shanghai, 200233 China; 3 Department of Tuberculosis Control, Wuhan Pulmonary Hospital, Wuhan, 430030 Hubei China

**Keywords:** Urethral stricture, Anastomosis, Pelvic fracture urethral distraction defect, Urethroplasty, Ancillary maneuver

## Abstract

**Objectives:**

Redo surgery for pelvic fracture urethral distraction defects (PFUDDs) is still a challenge. the long urethral defect makes it difficult while the high tension increase the recurrence rate. Although certain ancillary maneuvers can relieve tension, there is no consensus or guidelines for the prediction/planning of the selection. In this study, we present our experience with developing an intraoperative guidance system to achieve tension-free urethral anastomosis.

**Patients and methods:**

A total of 91 recurrent PFUDD patients managed at our center between 2020 and 2022 were retrospectively analyzed. The patients underwent scar removing and urethral anastomosis. For the long defect and high-tension cases, 6 kinds of tension-relieving maneuvers were used respectively during the process of urethral anastomosis. Preoperative assessment of the urethrogram was done before surgery, while biaxial (vertical and horizontal) defect measurements were performed intraoperatively. The patients were followed-up for 12 months (8.9 ± 4.2), furthermore, recurrence and complications were analyzed.

**Results:**

The overall success rate was 86.81%. The mean defect in urethrogram was 2.9 ± 1.1 cm. 27 simple anastomosis was performed when the vertical plus horizontal defect was less than 2 cm with 11.11% recurrence. 24 cavernous septum splittings were performed when the horizontal defect was greater than 2 cm with 8.33% recurrence. 21 inferior pubectomies were performed when the horizontal defect was greater than 3 cm with 19.05% recurrence. 15 ancillary distal urethra manipulations (fully distal urethral mobilization, urethral suspension and corpus cavernosa folding) were performed when the vertical defect was 3 to 4 cm with 13.33 recurrence. 4 reroutings were performed when the vertical defect was greater than 4 cm with 25.00% recurrence.

**Conclusions:**

Ancillary maneuvers are effective for reducing tension in redo urethral anastomosis. Measurement of divergent vertical and horizontal urethral defects could guide the selection of ancillary maneuvers. Combined tension-relieving maneuvers is recommended according to the defect direction and length to achieve a tension-free anastomosis.

**Supplementary information:**

The online version contains supplementary material available at 10.1186/s12894-024-01456-1.

## Introduction

A pelvic fracture urethral distraction defect (PFUDD) is one of the most common types of traumatic urethral stricture. The main treatment for PFUDD is anastomosis, which has not changed significantly in recent years compared to anterior urethroplasty [1–3]. The primary tension free end to end anastomosis have a high success rate because of relative normal anatomy, blood supply [4]. Redo PFUDD surgery is complicated by several anatomical features of the posterior urethral tissue, including a lack of corpus spongiosum to support the dissociative substitutes, the length of the path of the blood supply to the pedicle flap, compact scar tissue, and a deep location of the healthy proximal urethra [1–3, 5]. Although a bulbar membranous urethral anastomosis is commonly performed, defect > 4 cm are more complicated, especially in patients with multiple surgical failures, floating bladder, urethral fistula, or pediatric urethral strictures [1, 2]. The other problem is the discrepancy in defect length between that in the urethrogram and intraoperative measurement which causes the under estimation of the difficulty and complex during surgery.

Transperineal urethral anastomosis is the preferred surgical treatment for recurrent PFUDD; this procedure involves removing the atretic segment, exposing and trimming the distal and proximal urethral ends, and creating a tension-free urethral anastomosis [2, 3]. In general, the natural elasticity of the bulbar urethra allows the resection of a 1–2-cm-long segment to bridge a defect of similar length [6]. However, for patients with complicated long-segment stenosis, a large defect makes it difficult to achieve tension-free anastomosis due to stretching the distal and proximal ends, which leads to recurrence [7]. Treatment of a long posterior urethral stricture is challenging and requires the use of tension-relieving techniques. Opinions on the choice of technique are controversial [8]. Current guidelines and expert consensus statements published by the European and American urology societies recommend the use of evidence-based techniques for the treatment of posterior urethral strictures, but they do not provide detailed recommendations regarding the surgical procedures [9–12]. In addition to general treatment principles, guidelines should provide adequate details about the operation to perform urethral anastomosis. Previous studies have described several techniques to relieve the tension in anastomoses [13–17], but there is no consensus on the preferred technique. A conventional urethrogram does not predict the need for an inferior wedge pubectomy [8].

In previous studies, investigators have mostly described single methods for tension reduction. The aim of this study was to review patients with recurrent PFUDD underwent surgery in our center (the largest urethral center in China) in the past 2 years, to analyze various ancillary maneuvers and the circumstances for application. The research will provide a guidance system based on divergent biaxial measurement for tension free urethral anastomosis.

## Materials and methods

### Grouping criteria

**Fig. 1:**
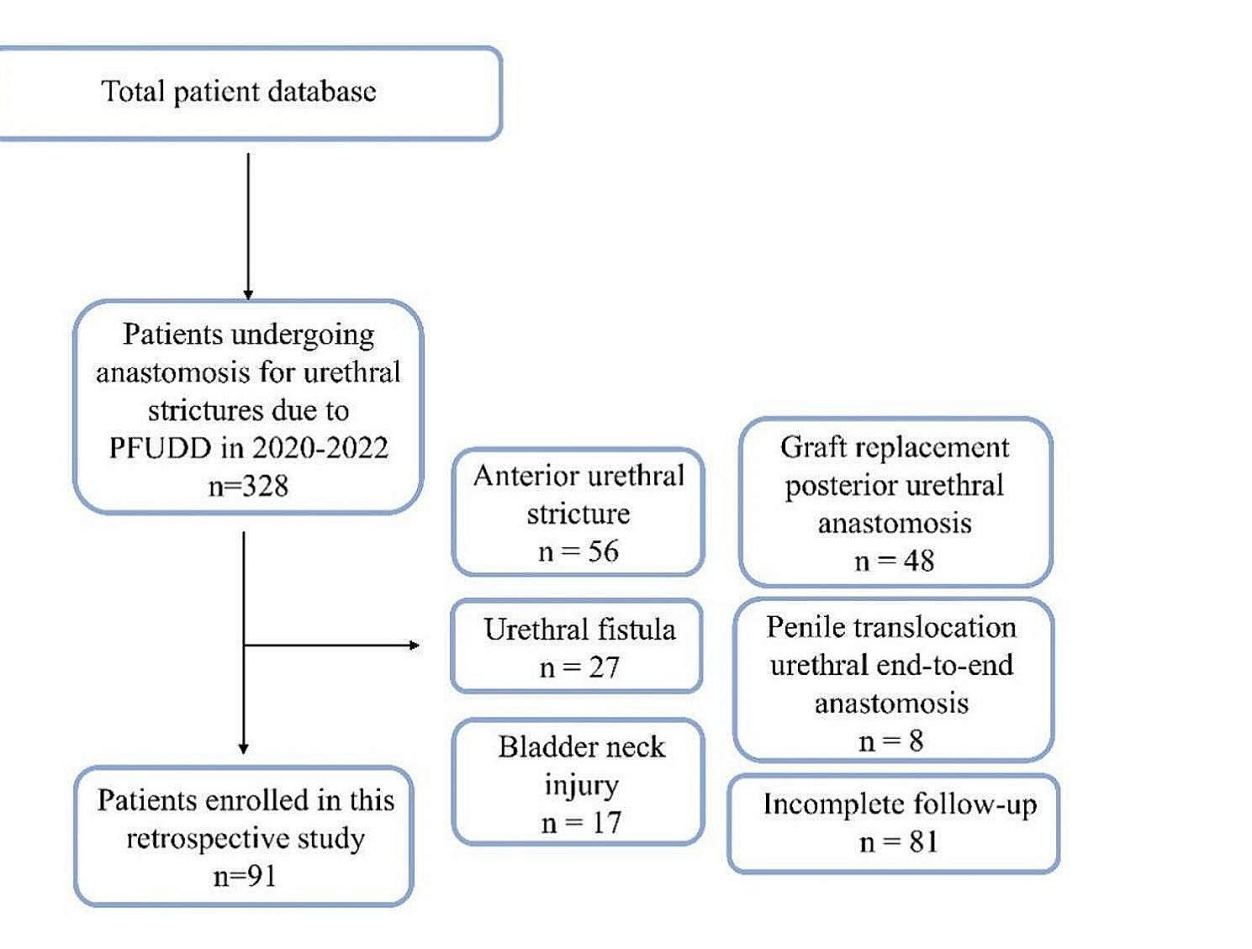
Retrospective chart review for inclusion and exclusion of cases

We retrospectively screened the records of 328 patients who underwent end-to-end urethral anastomosis at our hospital between January 2020 and December 2022 and selected 91 recurrent patients for inclusion in this study (Fig. 1). We selected patients with a 2–4-cm-long membranous urethral defect on urethrography that developed after a pelvic fracture who underwent suprapubic cystostomy at least 1 month before the operation. We excluded patients with a bladder neck injury (*n* = 17), urethral fistula (*n* = 27), accompanying anterior urethral stricture (*n* = 56), penile transposition urethral anastomosis (*n* = 8), graft replacement urethral anastomosis (*n* = 48), or incomplete follow-up (*n* = 81). The preoperative evaluation included age, time of injury, number and method of previous operations, physical examination, routine urine examination, urine culture, urinary flow rate, urethrogram, and urethroscopy. The patients underwent surgery 3 months after the pelvic fracture and had a lack of bacterial growth in urine cultures. Preoperatively, the suprapubic tube was replaced, and a urine culture was performed daily. Prophylactic antibiotics were administered according to drug sensitivity test results. The bladder was irrigated twice a day with 1,000 mL of 0.9% saline mixed with 5 mL of povidone-iodine. Surgery was performed after a negative urine culture result was returned. The patient was admitted to the hospital on the day of surgery.

### Surgical techniques

The patients were administered general anesthesia and placed in the lithotomy position. An inverted Y-shaped incision was made in the perineum, and the bulbous urethra was separated to free the distal and proximal ends. Then, the distal end was freed to the level below the scrotum and transected at the disrupted site of the membranous urethra. The scar tissue was removed to expose the mucosa on both ends of the anastomoses. Then, the vertical and horizontal urethral defect lengths were measured. The extension line of the distal urethra is the Y line, the extension line of the proximal urethra is the X line. The reference point is the end of distal urethra and the proximal urethra. The vertical distance is from the distal reference point to the X line. The horizontal distance is from the proximal reference point to the Y line. For patients with long defect and high tension, 6 ancillary maneuvers were performed successively (Video 1).

**Fig. 2:**
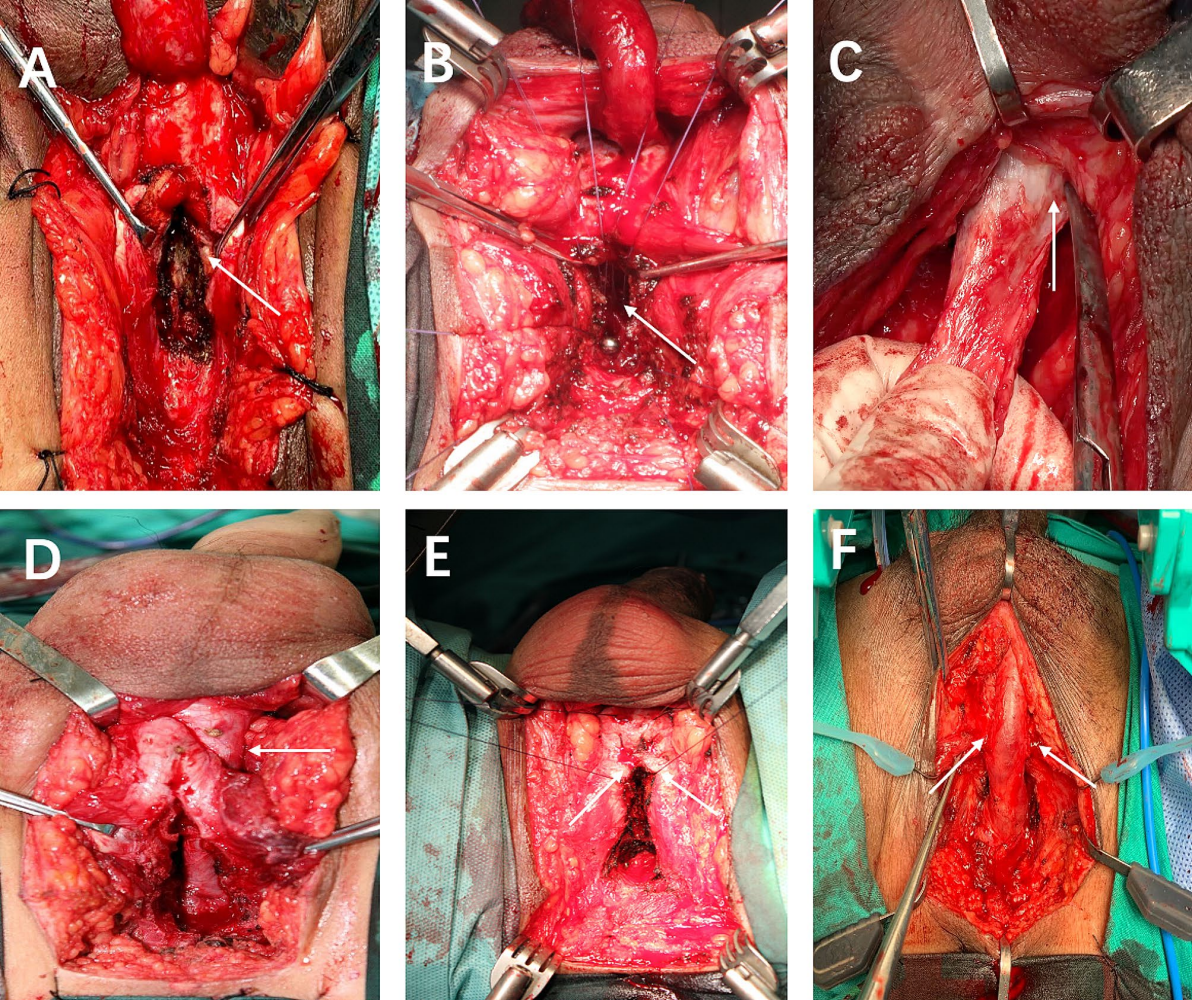
Ancillary maneuvers. A: penile cavernous septum incision; B: resection of the lower margin of the symphysis pubis; C: distal urethra mobilization; D: transposition of the anterior urethra at the foot of the cavernosum; E: folding of the penile cavernous body; F: urethral spongiform suspension

Cavernous septum splitting (Fig. 2A): An electric scalpel was used to incise the septum of the penile cavernous body along the middle of the junction. The depth of the septal cut reached the lower edge of the pubis. The distal urethra was embedded into the penile cavernous septum after anastomosis.

Inferior pubectomy (Fig. 2B): An electric scalpel was used to incise the periosteum to the lower edge of the pubic symphysis. Then, a wedge-shaped section of the bone was removed with a bottom width and height of 1.5–2.0 cm to create a direct urethral anastomosis with an osteotome.

Urethral mobilization to the penile-scrotal junction (Fig. 2C): Urethral tension was assessed by pulling the distal urethra to meet the proximal urethra. In the case of significant tension in the distal urethra, the urethra was freed to the penile-scrotal junction.

Rerouting of the anterior urethra (Fig. 2D): A channel was created to allow the anterior urethra to cross the tissue surrounding the bifurcation of the corpus cavernosum. Then, the anterior urethra was translocated to the lower edge of the corpus cavernosum to create a tension-free anastomosis with the posterior urethra.

Corpus cavernosa folding (Fig. 2E): This technique affects erectile function and should be avoided in patients who prefer to retain sexual function.

Urethral cavernous tissue suspension (Fig. 2F): Urethral tension after anastomosis was reduced by applying sutures to connect the two sides of the urethral cavernous tissue and the albuginea of the corpus cavernosum. The suture was placed on the corpus cavernosum 0.5 cm below that on the urethral cavernous tissue.

An F16 urinary catheter remained in place for 4 weeks after the operation, and the cystostomy tube was removed 3 weeks after normal urination resumed. Urine flow rate and urethroscopy were performed postoperatively.

### Follow-up

The postoperative data included recurrence of the stricture and time of recurrence. In the case of urinary incontinence, the method used to treat the incontinence was recorded. Letters, emails, medical software, and the social media application WeChat were used to record postoperative urine flow rates and complete self-report questionnaires, PROM (USS-PROM). Surgical failure was evaluated using the annual Qmax, which required a uroflow voided volume of at least 200 mL. For patients with a Qmax less than 15 mL/s, follow-up cystoscopy was performed. Failure of passage of an F16 flexible cystoscope was used to indicate stricture recurrence. The International Index of Erectile Function (IIEF) was used to evaluate sexual function before and after surgery, with scores less than 21 indicating erectile dysfunction (ED).

### Statistical analysis

SPSS software (version 22.0; IBM Corp., Armonk, NY, USA) was used for statistical analysis. The baseline characteristics were analyzed using descriptive statistics and are presented as numbers and percentages. The chi-square and Fisher’s exact tests were used to compare data. P values < 0.05 were considered to indicate statistical significance.

## Results

Table 1 presents the baseline information of the patients. The average age of the patients was 43.7 ± 13.2 years. In total, 6 (6.59%), 74 (81.31%), and 11 (12.09%) patients were aged < 18, 18–60, and > 60 years, respectively. The mean stricture length on the urethrogram was 2.9 ± 1.1 cm. The intraoperative mean horizontal and vertical defect lengths were 3.2 ± 1.3 cm and 3.5 ± 0.7 cm, respectively, indicating that urethrography seemed to be inefficient in the assessment of stricture length. Postoperatively, the average follow-up duration was 8.9 ± 4.2 months, and recurrence occurred at 3–6, 7–9, 10–12 and > 12 months in 27 (29.67%), 27 (29.67%), 26 (28.57%), and 11 (12.09%) patients, respectively.

**Table 1:** Descriptive analysis of the basic information (*N* = 91)

Variables	N (%)	Mean ± SD/median (quartile)
Age (years)		43.7 ± 13.2
< 18	6 (6.59)	
18–60	74 (81.31)	
> 60	11 (12.09)	
Intraoperative vertical defect length (cm)	91 (100)	3.2 ± 1.3
Intraoperative horizontal defect length (cm)	91 (100)	3.5 ± 0.7
Defect measured by urethrography (cm)	91 (100)	2.9 ± 1.1
Defect measured in operation (cm)	91 (100)	4.9 ± 1.3 *(p = 0.00)*
Number of days in hospital (days)		13.0 ± 5.1
<8	9 (9.89)	
8–14	59 (64.84)	
>14	23 (25.27)	
Follow-up (months)		8.9 ± 4.2
3–6	27 (29.67)	
7–9	27 (29.67)	
10–12	26 (28.57)	
>12	11 (12.09)	

Table 2 describes the choice of surgical approach and reduction strategy at different vertical and horizontal lengths of the urethral defect measured urographically and intraoperatively. Twenty-seven patients (29.67%) underwent direct anastomosis alone with a mean horizontal defect of 1.4 ± 0.5 cm, of whom 3 (11.11%) experienced recurrence at follow-up. Three patients (3.30%) underwent direct anastomosis + distal urethral-assisted reduction (distal urethral freeing, urethral suspension, penile corpus cavernosum folding) with a mean horizontal defect of 1.7 ± 0.6 cm and no cases of recurrence. Two patients (2.20%) underwent a simple cavernous septal split with a mean horizontal defect of 2.6 ± 0.6 cm. Nineteen patients (20.88%) underwent a cavernous septal split + distal urethral-assisted reduction with a mean horizontal defect of 2.7 ± 0.5 cm, with 1 recurrence (5.26%). Twenty-one patients (23.08%) underwent inferior pubectomy alone with a mean horizontal defect of 3.7 ± 0.8 cm, with 4 recurrences (19.05%) at follow-up. Fifteen patients (16.48%) underwent inferior pubectomy + distal urethral-assisted reduction with a mean horizontal defect of 3.7 ± 0.7 cm, with a total of 2 patients (13.33%) experiencing recurrence. Four patients (4.40%) underwent inferior pubectomy + distal urethral-assisted reduction + urethral rerouting with a mean defect of 3.9 ± 0.6 cm, with a total of 1 patient (25.00%) experiencing recurrence.

**Table 2:** The anastomosis and manipulation in regard to the divergent defect length

Anastomosis and maneuvers	N (%)	Mean ± SD/median (urethrogram length)	Mean ± SD/median (vertical length)	Mean ± SD/median (horizontal length)	Recurrence N (%)
Simple anastomosis	27 (29.67)	2.0 ± 0.5	2.4 ± 0.4	1.4 ± 0.5	3 (11.11)
Simple anastomosis + distal urethral manipulation	3 (3.30)	2.4 ± 0.3	3.1 ± 0.7	1.7 ± 0.6	0 (0)
Cavernous septum splitting	2 (2.20)	3.0 ± 0.6	2.7 ± 0.2	2.6 ± 0.6	1 (50)
Cavernous septum splitting + distal urethral manipulation	19 (20.88)	2.9 ± 0.4	3.3 ± 0.4	2.7 ± 0.5	1 (5.2)
Pubectomy	21 (23.08)	3.5 ± 0.2	2.9 ± 0.5	3.7 ± 0.8	4 (19.05)
Pubectomy + distal urethral manipulation	15 (16.48)	3.3 ± 0.4	3.9 ± 0.6	3.7 ± 0.7	2 (13.33)
Pubectomy + distal urethral manipulation + rerouting	4 (4.40)	3.4 ± 0.6	4.6 ± 0.3	3.9 ± 0.6	1 (25.00)

The data from the univariate analysis (Table 3) showed that although the difference between the age of the patients and the recurrence rate was not significant, this may have been influenced by the sample size and the survival period of the patients. The tabulated data indicate that, different from the conventional perception, the percentage of recurrence of urethral strictures was relatively high in patients < 18 years old, with a recurrence rate of 16.67%; a total of 10 patients between 18 and 60 years of age experienced recurrence, a rate of 13.51%, and only 9.01% of patients older than 60 years of age experienced recurrence. The statistical results showed that the presence or absence of a history of repair surgery prior to anastomosis was a relevant factor in the recurrence of urethral stricture and that patients with a history of surgery were more likely to have stricture recurrence than those without a history. One recurrence occurred in patients with a total length of stay < 8 days (11.11%), 8 recurrences (13.56%) in patients who stayed 8–14 days, and 3 recurrences (13.04%) in patients who stayed > 14 days; statistical analysis showed that length of stay was not a relevant factor for stricture recurrence.

**Table 3:** Univariate analysis of stricture recurrence and basic information

	Stenosis recurrence		Recurrence rate (%)	P value
	Yes	No		
Age (years)				>0.05
< 18	1	5	16.67	
18–60	10	64	13.51	
> 60	1	10	9.01	
Tension reduction method				>0.05
Pubectomy	4	17	19.05	
Pubectomy + distal urethral manipulation	2	13	13.33	
Pubectomy + distal urethral manipulation + Rerouting	1	3	25.00	
Number of days in hospital (days)				>0.05
< 8	1	8	11.11	
8–14	8	51	13.56	
> 14	3	20	13.04	

As shown in Table 4, mild SUI occurred in 1 patient (1.10%) after surgery. The overall recurrence rate of urethral restenosis was 13.17% (12 cases). The time to recurrence was distributed between < 6 months (5; 41.67%), 6–12 months (4; 33.33%), and > 12 months (3; 25.00%). Seventy-three patients had preoperative and postoperative ED data. Preoperatively, 46 patients (63.01%) reported ED, while 27 patients (36.99%) were able to achieve erection, of whom 19 patients (70.37%) had normal erections and 8 patients (29.63%) achieved only partial erections and were unable to have intercourse. After urethroplasty, 10 (37.03%) of the 27 patients with normal preoperative erections reported ED. Five (29.41%) of the 17 (62.96%) patients with normal postoperative erections reported mild erectile dysfunction.

**Table 4:** Recurrence and complications of urethral strictures after surgery

	N (%)	Mean ± SD/median (quartile)
Stricture recurrence		
Yes	12(13.17)	
No	79(86.81)	
Time to recurrence (months) (*n* = 12)		6.00(3.00–11.00)
< 6	5(41.67)	
6–12	4(33.33)	
> 12	3(25.00)	
Urinary incontinence		
No	90(98.90)	
Yes	1(1.10)	
Preoperative ED status		
No	27(36.99)	
Normal erectile function	19(70.37)	
Can get an erection, but cannot complete intercourse Chordee	8(29.63) 10 ( 37.04 )	
Yes	46(63.01)	
Postoperative ED status (only those without ED before surgery were counted)		
No	17(62.96)	
Normal erectile function	12(70.59)	
Can get an erection, but cannot complete intercourse	5(29.41)	
Yes	10(37.03)	

**Fig. 3:**
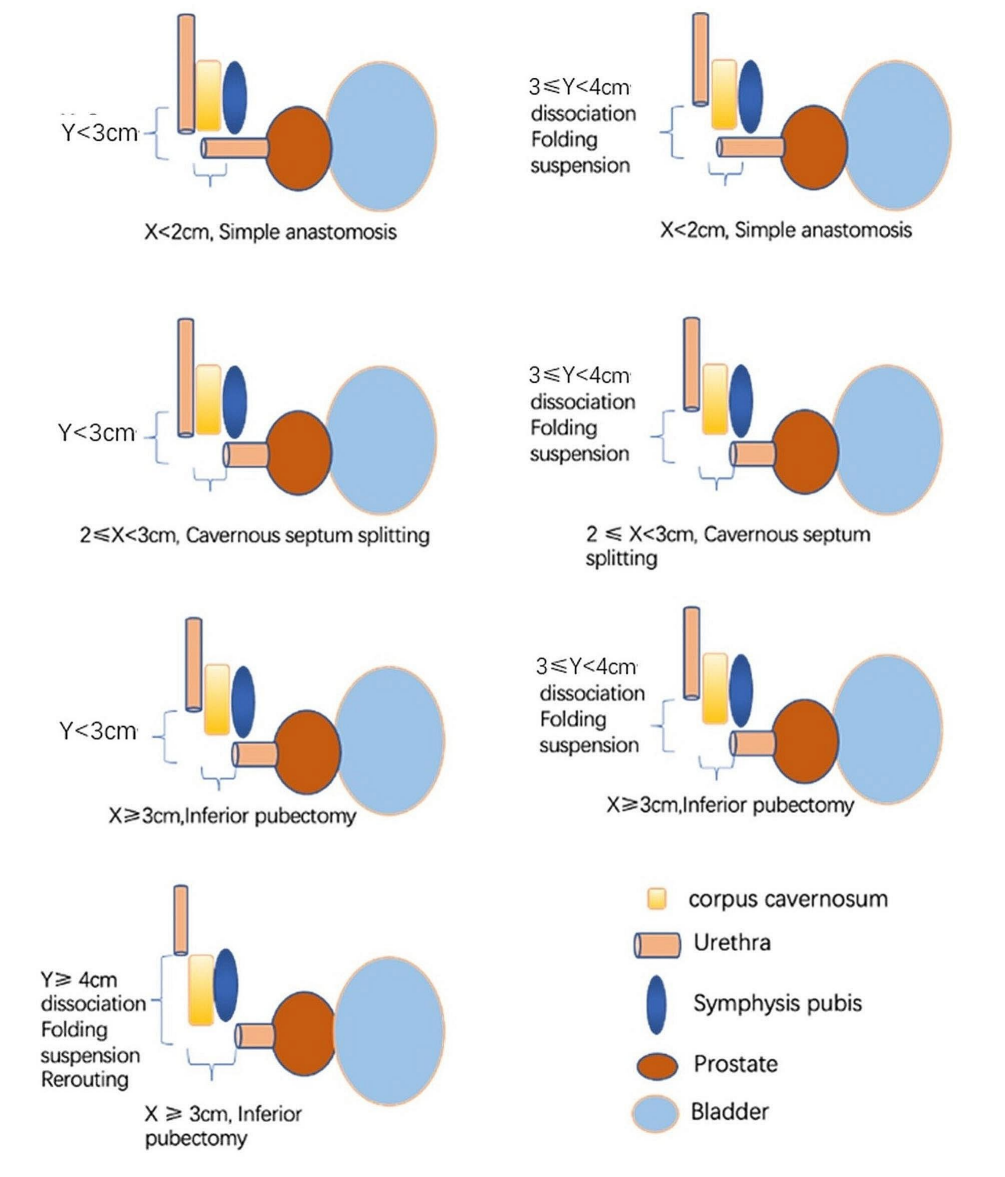
Schematics of the tension-relieving techniques according to the divergent defect length

We summarized a guidance system to decide when to perform a simple anastomosis or any of six additional tension-relieving maneuvers (Fig. 3). When the distance from the proximal urethra to the corpus cavernosum extension (horizontal defect length) was longer than 2 cm, the cavernous septum was dissected before the anastomosis was created; when it was longer than 3 cm, a pubectomy was performed. When the distance from the horizontal extension of the distal urethra to the distal end (vertical defect length) was shorter than 3 cm in the resting state without traction, a simple anastomosis was performed. When the vertical defect length was longer than 3 cm, distal urethral manipulations (distal urethral mobilization to the penile-scrotal junction, urethral suspending, and cavernosa folding) were performed. When the vertical defect length was 4 cm, urethral rerouting was performed.

## Discussion

In this study, we described 91 recurrent PFUDD patients who underwent redo urethral anastomosis at our center over the past 2 years. The urethrogram is the most popular method for urethral evaluation, which could provide the condition/length of anterior urethra, gap condition, degree of malalignment and bone related factors. Recently, we noticed that there was a significant difference between the urethrogram and intraoperative measurement of the urethral defect length. Therefore, urethrograms are not adequate for the determination of urethral stricture complexity and the need for ancillary manipulations. We performed different surgical procedures to reduce the tension in anastomoses and measured divergent urethral defect lengths on vertical and horizontal directions. In the results, we introduced a guidance system to apply ancillary maneuvers.

Releasing the entire distal urethra as far as the suspensory ligament of the penis is necessary to create a tension-free anastomosis and results in an increase of almost 3 cm in urethral length [18]. Chordee formation can be prevented by avoiding excessive dissection beyond the suspensory ligament of the penis. Koraitim reported that 73% of patients were treated with urethral mobilization alone, and simple surgery was adequate for patients with a defect shorter than one-third of the bulbar urethral length on the urethrogram [14]. In Western populations, the urethra is longer and can be stretched to achieve a simple tension-free anastomosis [8]. However, Flynn [16] reported that among 120 patients treated with perineal posterior urethroplasty, only 8% required urethral mobilization alone. In our study, 27 patients (29.67%) underwent a simple anastomosis and had a low recurrence rate (11.11%).

Corpus cavernosum septum splitting is frequently performed to relieve tension in urethral anastomoses, although the depth of the incision varies. In Flynn’s study [16], 34% of patients required corporal splitting, compared to 23.08% of patients in our study. For most pelvic fractures and urethral strictures, corpus cavernosum septum splitting is adequate to fully expose the proximal anastomosis. However, it also reduces the urethral length required and changes the acute angle of the anastomosis to an obtuse angle, which reduces the tension of the anastomosis.

For patients with a long defect due to the bladder ascending in the horizontal direction, an inferior pubectomy is needed. The need for inferior pubectomy cannot be predicted. Andrich et al. [19] reported no association between the measured defect length and type of subsequent surgery in 62% of patients. PFUDD severity and the need for inferior pubectomy are much higher in Asian countries (67% in India) than in Western countries (18% in Italy and 10% in the US) [15, 16, 20, 21]. Due to the complexity of the cases referred to our hospital, the proportion of patients who required an inferior pubectomy was high. Kulkarni et al. [22] reported that inferior pubectomy was inadequate in many second urethral anastomoses, which led to surgical failure.

Supracrural rerouting is a controversial maneuver performed by Webster in 11 cases; the need for this maneuver cannot be predicted preoperatively [13, 18]. In a previous study, 66 complex cases (4.12%) underwent supracrural rerouting, most of whom were underage [17]. This method was rarely used in other studies; supracrural rerouting was performed in only 2.8% of patients in Kizer’s report [15] and not at all in Morey’s report [13]. Saini et al. [23] suggested that rerouting adds 2 cm of urethral length for the anastomosis. In this study, paracavernous bypass was used in 4 patients, most of whom were preadolescent patients.

The true incidence of ED after PFUDD surgery is difficult to estimate. First, preinjury erectile function is usually not well documented with objective findings. Almost all data on pre-PFUDD erectile function are described by patients after the onset of trauma by subjective recall and are therefore greatly influenced by recall bias. For this reason, there is also substantial variation in the literature regarding the reporting of ED rates after PFUDD. ED rates are not only variable but also subjective and often based on patient expectations of treatment outcomes. The true incidence of ED after PFUDD could be better elucidated if these data were more effectively and objectively collected to obtain robust evidence.

The cause of recurrence remains elusive as of now. We hypothesize that factors such as anastomotic tension, compromised blood supply resulting from excessive mobilization of the distal urethra or bulbar artery injury, residual scarring at the urethral ends, as well as postoperative inflammation or infection could be potential contributors. Ultrasound is frequently employed to evaluate the length and scarring of the urethra, which we believe could serve as a valuable tool in predicting recurrences. However, we have not utilized penile Doppler to assess the adequacy of blood supply. In the future, it could be a method to predict the failure and recurrence.

There are still some limitations in this study. Firstly, this is a retrospective study with recall bias and selection bias. The patients underwent prior failed surgery which influenced the base line. Secondly, there is no data about the prior failed procedures, because the patients come from different hospitals of China. In China, the patient’s data is still not opened to every hospital, so we can not get the data. All the patients have to get examinations including urethrogram in our hospital. At last, the sample size is small and more prospective, randomized studies are needed in the future.

## Conclusion

The urethral defect length measured on a traditional urethrogram differed significantly from that measured intraoperatively. To achieve a tension-free anastomosis, multiple combined tension-relieving maneuvers were recommended according to the defect’s direction and length. Our results support the use of divergent vertical and horizontal urethral defect lengths for the prediction of difficulty in PFUDD treatment and the need for ancillary maneuvers in transperineal urethral anastomosis.

### Video legend

The measurement method of divergent vertical and horizontal urethral defect lengths and six tension-relieving maneuvers.

### Electronic supplementary material

Below is the link to the electronic supplementary material.

Supplementary Material 1

Supplementary Material 2

## Data Availability

The data that support the findings of this study are available from Shanghai Eastern Institute of Urologic Reconstruction but restrictions apply to the availability of these data, which were used under license for the current study, and are not publicly available. Data are available from the authors upon reasonable request, and with permission of Shanghai Eastern Institute of Urologic Reconstruction.

## Data availability

All the data and materials are available.
