# Supplementary material for: Retrospective analysis of urethral anastomosis with ancillary maneuvers and intraoperative biaxial defect measurements to achieve a tension free guidance system for redo PFUDD treatment
Source: BMC Urol. 2024 Apr 9;24:82. doi: 10.1186/s12894-024-01456-1 (PMC11003013; doi:10.1186/s12894-024-01456-1)
Supplement: Supplementary file 2 — Supplementary Material 2 [file 12894_2024_1456_MOESM2_ESM.docx]

Supplementary Video1: Intraoperative biaxial defect measurement for guiding urethral anastomosis with ancillary maneuvers
